# Exploring areas of improvement in postnatal care services in a tertiary care hospital in Lahore

**DOI:** 10.12669/pjms.39.3.6780

**Published:** 2023

**Authors:** Atiya Mahmood, Amna Zia Eusaph, Ayesha Javed, Arooj Muzaffar

**Affiliations:** 1 Dr. Atiya Mahmood, MBBS. Medical Officer, Government Samanabad Hospital, Lahore, Pakistan; 2 Prof. Dr. Amna Zia Eusaph, MBBS, FCPS. Lady Aitchison Unit 5, Lahore, Pakistan; 3 Dr. Ayesha Javed, MBBS. Post Graduate Trainee, Mayo Hospital, Lahore, Pakistan; 4 Dr. Arooj Muzaffar, MBBS. Post Graduate Trainee, Mayo Hospital, Lahore, Pakistan

**Keywords:** Postnatal care quality, Obstetric care in Pakistan, Puerperium improvement

## Abstract

**Background and Objective:**

Poor postnatal care can increase morbidity and mortality. This study assessed the current deficiencies in quality of postnatal care provided to mothers in Lady Aitchison hospital, Lahore when compared to WHO standards and identifies the areas for quality improvement.

**Methods:**

It is a descriptive cross-sectional study which employs quantitative method to collect and analyze the data. It was conducted to include ninety-six maternities attending the wards of Lady Aitchison Hospital, Lahore during January 2022 to February 2022. Consenting post-partum mothers were selected by random sampling and interviewed by using a structured proforma.

**Results:**

Among 96 mothers, 56% were below 25 years of age, 39% had secondary education, with more than one child (71%) and 57% visited for the first time. Majority of mothers, were given medicine timely (82%) and found the attitude (85%) and information (83%) provided by the healthcare workers helpful. Their subjective satisfaction rate with staff was 90%. The main areas of concern were lack of proper examination guidelines and facilities, limited information to mothers regarding neonatal care and substandard interior of hospitals. The statistics on the detailed maternal and neonatal examination showed that it was left out in 30% to 50% patients. Information regarding the danger signs of mothers and neonates was not given in 69% and information on family planning was provided to only 28%. Contentment with the infrastructure of the hospital was subpar and it was suggested that the sanitary conditions of washrooms and the paraphernalia of the wards i.e., ACs and beds needed improvement.

**Conclusions:**

This study suggests that in developing countries like Pakistan, majority of the patients were satisfied by the services of healthcare workers. The prime improvement area is the infra-structure of the hospital which can be upgraded to provide better quality facilities in terms of air-conditioning, washrooms and well-designed areas for extensive examination of breast, pelvis, abdomen and neonates. There is also need for introduction of standard guidelines for postnatal care.

## INTRODUCTION

Postnatal care services consist of care given to mother and her newborn for the first six weeks following birth. WHO describes this period as the most dangerous for mother and newborn. Almost half of the maternal deaths occur within the first 24 hours and 66% during the first week.^1^ Poor care in this time period can increase morbidity and mortality. Globally, 295,000 women died due to complications of pregnancy and childbirth in 2017.^2^ Developed countries have an estimated maternal mortality rate of 8-16 per 100,000 live births annually whereas in developing countries the estimate is 237 per 100,000 live births (WHO).^3^ MMR in Pakistan is 186 per 100,000 live births as estimated in 2019.^4^ Maternal mortality is high in Pakistan due to shortage of doctors, nurses and beds at government hospitals.

Research on existing facilities and subsequent audit is the key to identifying improvement areas and subsequently to reduce MMR. A research paper from Dedza district, Malawi.^5^ stated that human and material resources for provision of comprehensive and quality postnatal care services were inadequate. Another research from Mbeya district.^6^ concluded that structure and process for provision of quality PNC had gaps and has not been implemented as per guidelines. There were no separate rooms with all facilities, lack of privacy and confidentiality, inadequate infrastructure, insufficient healthcare providers as compared to private hospitals where separate postnatal unit and rooms, high midwife to women ratio and skilled staff was available.

According to study conducted in Gambia^7^ there were inadequacies in blood transfusion, essential medicines, accompanied by poor staff attitude and delayed provision of cesarean, inadequate government funding, poverty of patients and shortage of doctors. In India^8^ 39% mothers were moderately satisfied, 60% minimally satisfied and 1% satisfied by services provided by nursing personnel. In Iran^9^ mothers were satisfied with the facilities but less satisfied with workers.

A research paper from central Shanghai, China^10^ demonstrated that mothers were unsatisfied due to decrease education, training and unavailability of health personnel in emergency conditions. In Kenya^11^, the introduction of a comprehensive care package in mothers improved the quality of postnatal counseling about infant breast feeding and family planning and upgraded the maternal care index to about 8.72 out of 23. Peer support produced significant reduction in postnatal depression in high risk women.^12^ Proper educational programs are helpful in reducing unplanned pregnancies.

To address the high MMR in Pakistan, there is a dire need to observe the areas of improvement in postnatal care services. Hence, the current study was conducted with the objective of exploring in detail the three elements: structure, process and outcome^13^ of maternal healthcare in Lady Aitchison Hospital. This research will help the authorities to formulate the policies to improve health services in developing hospitals.

## METHODS

The study design was cross-sectional descriptive in which quantitative method was used to collect and analyze the data. The study used a questionnaire which was filled by 96 patients at Lady Aitchison hospital, Lahore from January 2022 to February 2022 (No.226/RC/KEMU). A questionnaire was designed in the form of three-page proforma and distributed randomly. After approval of Institutional Review Board (IRB) KEMU, Lahore, administrative permission from authorities of all relevant hospitals was obtained. Prior to collection research design was explained to the physician on-call. Consent was taken before asking question. Questions were explained to the mothers and filled out in the questionnaire based on Donabedian’s^13^ (2005) model. Patient-doctor interaction was not intervened. The patients were selected through simple random sampling.

### Sample Size:

Sample size of 96 patients was estimated by using 95% confidence level, 10% absolute precision with expected percentage postnatal care services as 48%.


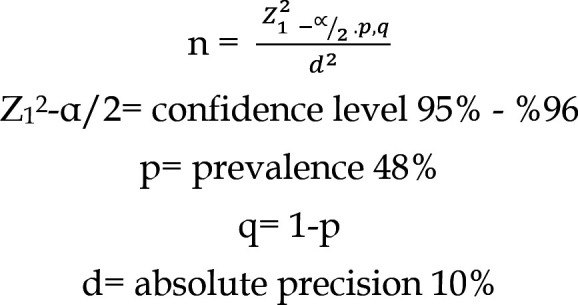


Strict inclusion criterion to include consenting mothers receiving postnatal care services within 48 hours after deliver was applied and non-consenting mothers and follow-up cases were excluded.

### Data Analysis:

Data was done by entering it in SPSS-22. Quantitative variables like age will be presented as mean SD. Qualitative variables like gender will be represented as frequency and percentage.

### Ethical Considerations:

All ethical issues such as maintaining confidentiality and avoiding harm were strictly observed during the study.

## RESULTS

The demographic profile of the mothers who were assessed for the postnatal care at Lady Aitchison hospital showed distribution in age, socioeconomic status, education level and number of visits and number of pregnancies. These are shown in Table-I. Out of 96 mothers, 56% were between 20-25 years of age, 30% between 26-30 years remaining being above 30. Total 76% mothers had family income greater than 10,000. Regarding education, 23% had primary, 39% had secondary, 21% had higher education and 17% were illiterate. About 57% mothers visited Lady Aitchison hospital for the first time and 26% for the second time. 29% mothers became pregnant for the first time, 34% for the second, 11% for the third, 14% for the fourth, 8% for the fifth and 4% for sixth time.

**Table-I:** Shows demographic data of study population.

Demographic Data	Frequency(%)
Age	
20-25	56
26-30	30
31-35	13
36-40	1
Income	
<1000	76
>1000	24
***Gravidity***	
1	29
2	34
>2	36
***Education Level***	
Primary	23
Secondary	39
Higher	21
None	17
***No. of Visit***	
1	57
2	26
>2	17

### Quantitative findings:

The satisfaction with the different elements of health care have been plotted in Table-II. The satisfactory areas of postnatal care included the healthcare workers’ performance and the availability of medicines. Eighty five percent said that the attitude of healthcare providers towards them was acceptable. Overall, 90% of mothers were satisfied with the quality of care by healthcare workers. As regard to availability of medicines, 95% mothers were given medicine timely.

**Table-II:** Shows elements of health care provided

Element of Care	Frequency (%)
***Subjective satisfaction***	
Sanitary Conditions	82
Medicine Administration	95
Attitude of Healthcare Professional	85
Quality of Care	90
***Maternal examination***	
BP	95
Pulse	84
Temperature	69
General Examination	75
Breast Examination	35
Abdominal Examination	66
Pelvic Examination	48
Wound Examination	63
***Neonatal Examination***	
General Examination	76
Temperature	63
Cord Examination	65
***Information Provided By Healthcare Professional***	
Danger Signs	31
Breast Feeding	80
Family Planning	28
Maternal Nutrition	70

The statistics on the examination done on patients showed it was sub-optimal keeping in view its significance on patients’ management. Although, blood pressure and pulse were taken in 95% and 85% of patients, the temperature was not checked in 31 % of mothers. The general, abdominal, breast and wound examination was also deficient. General examination was not performed in 25%, breast examination had not been done in 65%, abdominal examination in 34% and the pelvic examination in 51% mothers. Wound examination had been done in 63% but not in 30%. The examination on babies, showed that general examination of baby had been done in 76% respondents only.

The temperature and cord examination of 63% and 65% babies was done respectively. This reflects lack of proper facilities to perform examinations and the need for proper guidelines on examination and improvement in facilities e.g., increasing the staff number and provision of proper rooms so that healthcare providers can perform these examinations effectively. Attention must be paid to this area as these vital measurements can drastically improve quality of care.

Detailed information provided by healthcare workers was also deficient; information on maternal and infant danger signs was given in 31% respondents and left out in 69%. They were not told when to contact the doctors if they observed particular danger signs in their own and newborns. Similarly, information on family planning was given in only 28%. However, mothers agreed that information on breast feeding and maternal nutrition was given in 80% and 70% mothers respectively.

### Qualitative findings:

The mothers who weren’t satisfied were asked to mention areas of improvement. They mentioned that the infrastructure of the hospitals including ACs and washrooms needed to be improved. Many mothers were having problem with the sanitary conditions, though the hospital itself maintained quite a good state of cleanliness, its washrooms were not in a good condition and it was suggested by many mothers that hospital administration should pay more attention to the sanitation and proper water supply.

### Correlation:

Correlation between variables was calculated using chi-square test. It showed association among the following confounding variables:

- Age of respondents and the helpfulness of information provided by health care staff. (p=0.010)
- Age of respondents and the attitude of health care providers towards them. (p=0.002)
- Age of respondents and their satisfaction with the quality of care. (p=0.001)
- Education levels of the respondent with the attitude of health care providers towards them (p=0.009).

## DISCUSSION

Postnatal care services if provided appropriately can help reduce the maternal and infant mortality rates. Unfortunately, maternal and infant mortality rates are towards the higher end. During 1990 to 1999, average maternal mortality ratio at Lady Willingdon Hospital was 681 /100,000 live births. A study suggested that multi-sectoral coordination, poverty alleviation, improving socioeconomic status of women, their nutrition and general health, availability of good quality health services and adequate contraceptive/reproductive services were required to improve maternal health.^14^ In 2013-2017 a study in tertiary care hospital in Peshawar, showed that the maternal mortality during this period was 431/100,000 live births.^15^ This indicates that with healthcare reforms, maternal mortality rate is decreasing in Pakistan but it is still overbearing when compared to developed countries. To reduce this MMR key areas highlighted by our research are proper examination of temperature, general physical, abdomen, pelvis and wound along with neonatal general, temperature and cord examination. Secondly, detailed information on danger signs and breast feeding must be provided. Thirdly, administrative authorities should channel their funding to improve the washrooms, examination rooms and provide better air conditioning in the wards.

Our study showed that majority (95%) mothers said they were given timely medicines while 85% mothers found the attitude of health care worker to be pleasant. These results are similar to those in China^16^ where more than 80% mothers were satisfied with the politeness of health workers. Here, 90% of the mothers were satisfied in general with the quality of care provided to them at Lady Aitchison. Previously, in Pakistan^17^ 61% mothers who visited government hospitals were not satisfied. This shows there is improvement over the years co-relating with reduction in MMR. Maternal satisfaction is important because women who perceive quality of care to be low may avoid facility based care increasing the rate of maternal mortality and morbidity.^18^ Maternal expectations generally match the providers perspective of care^19^ and hence must be met vigilantly.

A study in Lilongwe district^20^ showed that less than 75% midwives inspected perineal wounds and checked neonatal and maternal vital signs. In our study, examination was deficient as maternal temperature was taken in only 69%, general examination in 75%, breast examination in 35%, abdominal examination in 66%, pelvic examination in 48% and wound examination in 63% mothers. Neonatal examination was done in 76%. Only, 63% and 65% said temperature recording and cord examination of newborn was done. These findings are inadequate when compared to developed healthcare set-ups.

According to WHO,^21^ a full clinical examination of mother and neonates should be done around one hour after birth. All postpartum women should have regular assessment of vaginal bleeding, uterine contraction, fundal height, temperature and heart rate (pulse) routinely during the first 24 hours starting from the first hour after birth. Blood pressure should be measured shortly after birth. If normal, the second blood pressure measurement should be taken within six hours. Urine void should be documented within six hours. Beyond 24 hours, micturition and urinary incontinence, bowel function, healing of any perineal wound, headache, fatigue, back pain, perineal pain and perineal hygiene, breast pain, uterine tenderness, lochia, breastfeeding progress, emotional well-being, resumption of sexual intercourse and possible dyspareunia should be assessed.

Newborn must be monitored for proper feeding, convulsions, fast breathing (breathing rate ≥60 per minute), chest in-drawing, no spontaneous movement, and altered body temperature (temperature ≥ 37.5°C or <35.5°C) any jaundice in first 24 hours of life, or yellow palms and soles at any age. Careful inclusion of these elements is required to improve MMR. Cord care with daily chlorhexidine is recommended at low quality postnatal care facilities.

A quality improvement study in Sweden showed woman did not receive information regarding specific postnatal aspects.^22^ In our study, information on danger signs and family planning was insufficiently provided. WHO^21^ guidelines state that key newborn care messages such as immunization, vulnerability of preterm and low-birth-weight babies, infant danger signs should be communicated to mothers. Mothers should be counselled to delay bathing the newborn until 6-24 hours after birth. Baby should have one to two layers of clothes more than adults, hats/caps must be used. The mother and baby should stay in the same room. Signs of postpartum haemorrhage (faintness, dizziness, palpitations/tachycardia), pre-eclampsia/eclampsia(headaches accompanied by one or more of the symptoms of visual disturbances, nausea, vomiting, epigastric or hypochondrial pain, feeling faint, convulsions), infection (fever, shivering, abdominal pain and/or offensive vaginal loss.) and thromboembolism (unilateral calf pain, redness or swelling of calves, shortness of breath or chest pain.), nutrition, hygiene, family planning, safer sex, use of insecticide-impregnated bed nets, gentle exercise must be reinforced at birth.

In government hospital of Xiengkhouan,^23^ research showed only 22% mothers were satisfied with sanitary facilities. In Nigeria^24^ lack of accept infrastructure and human resources was the cause of poor satisfaction among mothers. In Sindh,^25^ research showed that affordability and availability of services around pregnancy and birth were major factors responsible for preference for maternal care and could be linked with poor obstetrical care among rural women of Sindh.

Another study conducted in four provinces of Punjab,^26^ suggested that targeted skill-based training and provision of adequate drugs and equipment are required to improve the quality of Emergency Obstetric and Newborn Care. Improvement in these areas can bring about a potential reduction in maternal mortality. Our research interviewed mothers and concluded that they were not satisfied by the sanitary conditions.

Furthermore, a variety of confounding factors are involved in determining the quality of care. A service quality gap study^27^ conducted in public hospitals of Rawalpindi showed that satisfaction with quality was lower and association of service quality was statistically significant with gender, education, occupation, monthly income, and the number of visits to the hospital. Maternal satisfaction on post-partum care is mainly affected by residency, antenatal care follows up, mode of delivery, and complications during birth.^28^

Women with higher education are more likely to give birth in equipped facilities.^29^ In our research, it was found that age and education level of mother influenced the helpfulness of information provided by staff, the attitude of health care providers towards them and their satisfaction with the quality of care. Hence, multisector interventions are needed to decrease MMR. These confounding variables must be taken into account while assessing quality of care in hospitals.

### Limitations:

Our findings were confounded by the age and education level of mother. It can be used to conduct large scale studies which will remove the influence of these variables and will allow more clarity on the elements of consideration.

## CONCLUSION

Multiple areas of improvement including paraphernalia, infrastructure, examination areas, proper guidelines for examination, sanitary conditions, detailed information to patients have been observed. Substandard hospital sanitiation and air-conditioning can deteriorate the quality of healthcare services and need upgradation for overall amelioration of care. Moreover, healthcare workers must be provided with proper guidelines for examination and charts can be installed in hospitals to ensure detailed examination of mothers is done adequately. Women must be acquainted with sufficient information about danger signs of neonates to reduce mortality. Implementing an intensive postnatal care program will reduce the burden of maternal and neonatal mortality.

## Abbreviations:

**MMR:** maternal mortality rate, **WHO:** World Health Organization, **PNC:** postnatal care, **SD:** standard deviation, **SPSS:** statistical package for social sciences, **IMR:** infant mortality rate, **B.P:** blood pressure.
